# Long-term outcomes in health-related quality of life influence chronic disease management in patients with pulmonary hypertension

**DOI:** 10.3389/fcvm.2022.1008253

**Published:** 2022-11-10

**Authors:** Jin-Ling Li, Fan Xiao, Hong-Ting Liu, Hui-Ting Li, Qin-Hua Zhao, Chun-Yan Sun, Yan Zhu, Lei Yan, Wen-Yue Wang, Hui Luo, Su-Gang Gong, Rong Jiang, Jin-Ming Liu, Rui Zhang, Lan Wang

**Affiliations:** Department of Pulmonary Circulation, School of Medicine, Shanghai Pulmonary Hospital, Tongji University, Shanghai, China

**Keywords:** pulmonary hypertension, health-related quality of life (HRQOL), SF-36, long-term outcome, chronic disease management

## Abstract

**Background:**

Significantly improved survival in patients with pulmonary hypertension (PH) has raised interest in maintaining a good quality of long-term survivorship. In this study, health-related quality of life (HRQOL) measurement was used to assess the long-term changes of physical and mental outcomes.

**Methods:**

A total of 559 consecutive inpatients with PH completed generic HRQOL (Short Form-36) who were diagnosed with PH by right heart catheterization. Assessments were carried out at short-term (1 year), midterm (3 years), and long-term (5 years) follow-ups.

**Results:**

Patients with PH suffered more severe impairments in both physical and emotional domains than the U.S. population normative values. Patients with PH due to chronic lung disease had the worst physical component summary (PCS) score, but there was no difference in mental component summary (MCS) score among different PH types. A reduced PCS score was correlated with WHO FC severity and pulmonary vascular resistance (PVR). The Z score showed that the changing trend of mental conditions continuously declined from baseline to midterm and long-term follow-ups, but the PCS score seemed to be stable or improved. Cox regression analysis indicated increased baseline PVR and WHO FC III and IV, and decreased physical subscale of role physical, mental subscale of social functioning, and the MCS score have increased risk of mortality in the long-term follow-up.

**Conclusion:**

Patients with PH have poor HRQOL. The long-term change of physical status seemed to be stable, but the mental state was continuously worse. These suggested identifying and intervening mental health progresses is a noteworthy issue in PH chronic management.

## Introduction

Pulmonary hypertension (PH) is a pathophysiological disorder that involves multiple clinical conditions and complicates the majority of cardiovascular and respiratory diseases (1). According to 2015 European Society of Cardiology (ESC)/European Respiratory Society (ERS) guidelines for the diagnosis and treatment of pulmonary hypertension, PH can be categorized into mainly five groups based on their clinical conditions, pathological findings, and hemodynamic features and treatment strategy: pulmonary arterial hypertension (PAH, group 1), pulmonary hypertension due to left heart disease (LHD-PH, group 2), pulmonary hypertension due to lung disease and/or hypoxia (CLD-PH, group 3), chronic thromboembolic pulmonary hypertension and other pulmonary artery obstructions (CTEPH, group 4), and pulmonary hypertension with unclear and/or multifactorial mechanisms (1, 2). Advances in medical therapy and surgery have improved symptoms, function capacity, and survival of patients with PH, particularly with PAH and CTEPH to varying degrees (3, 4). However, the outcome and survival of patients with PH are still not satisfactory, which are essential for the management of patients with PH.

Over the past two decades, clinical research in cardiopulmonary disease has broadened from a physiological base to a more comprehensive approach of the health-related endpoint (5). The Medical Outcomes Survey Short-Form 36 (SF-36), a patient-reported outcome measure, is an important tool for assessing health-related quality of life (HRQOL) (6). Most studies in PH focused on short-term changes in etiology of PAH and CTEPH, typically as response to therapies or secondary outcomes in clinical trials. To our knowledge, patients with PAH and CTEPH experience significantly worse HRQOL than the general U.S. population (7–10). Previous work on 55 patients with PAH demonstrated that the physical component summary (PCS) and mental component summary (MCS) scores of their HRQOL were poor, mainly in the physical functioning (PF) domain, less so in the psychological functioning domain (10, 11). Similarly, individuals diagnosed with CTEPH scored less in nearly all SF-36 parameters (12). Decreases in mental health parameters are more pronounced in CTEPH than in PAH (13, 14). On the other hand, PAH treatments have been proven to improve HRQOL, which correlates with better exercise capacity, functional class, or hemodynamics (11, 15).

However, long-term information about quality of life, functional result, and mental changes in PH is still limited. The main focus of this study was to gather data from short-term, midterm, and long-term cohorts of survivors of PH by measuring generic HRQOL (SF-36). The secondary objective was to analyze the changing trend of HRQOL from baseline to long-term follow-up. Finally, we explored the prognostic risk factors associated with long-term mortality among physical and mental components.

## Materials and methods

### Patients and study design

We consecutively enrolled adults patients with PH (≥18 years of age at diagnosis) with an established diagnosis of PH in Shanghai Pulmonary Hospital from November 2010 to January 2018. PH is defined by a mean pulmonary artery pressure (mPAP) of ≥ 25 mmHg at rest, measured by right heart catheterization (RHC) (1, 16). Clinical classification of PH in this study was considered according to guideline standards, mainly including PAH, LHD-PH, CLD-PH, CTEPH, and others (1). The baseline assessment at the time of diagnosis included medical history, physical examination, 6-min walking distance (6MWD), World Health Organization (WHO) functional class, N-terminal fragment of pro-brain natriuretic peptide (NT-ProBNP), and hemodynamics. The study conformed to the principle of the Declaration of Helsinki and was approved by the Ethics Committee of Shanghai Pulmonary Hospital (Number: K16-293). Written informed consent was obtained from all patients.

**Figure 1:**
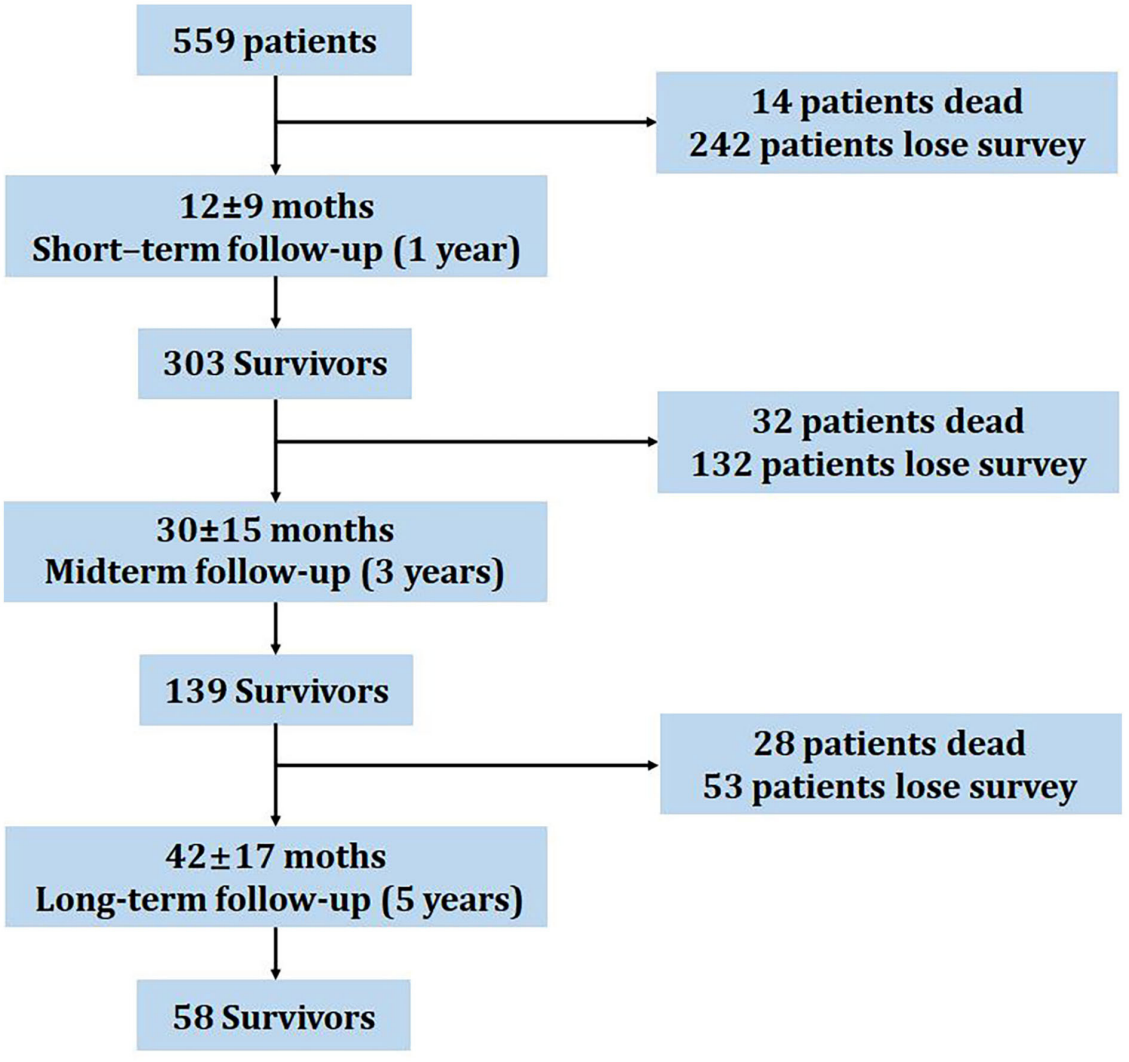
Study patient flow diagram.

Figure 1 describes the patient flow. A total of 559 patients with PH initially completed self-report measures of generic HRQOL who were diagnosed with PH by RHC. Patients completed questionnaires during the follow-up. During the follow-up, survivors who were not able to return the questionnaires or dead patients were excluded from the study. After a mean 12 ± 9 month-interval follow-up (short-term), 32 patients (4%) died and 132 patients (24%) were lost to follow-up. Accordingly, the remaining 303 surviving patients had a second follow-up after 30 ± 15 months (midterm). The last survey was completed after 5 years (42 ± 17 months, long-term) from the remaining 139 survivors. Patients with any mental disorder who could not understand and fill out the questionnaires by themselves were excluded from the study.

### Study measurement

General HRQOL was assessed using the Medical Outcomes Study 36-item Short-Form Health Survey (SF-36, version 2) in all patients (17). It consists of eight scale domains: physical functioning (PF), role physical (RP), body pain (BP), general health (GH), vitality (VT), social functioning (SF), role emotion (RE), and mental health (MH) (18). A score for each domain is provided between 0 and 100, with 100 being the best possible health and 0 the worst possible health. These domains can yield two summary scores for physical component summary (PCS) and mental component summary (MCS). Each domain and summary score was standardized against a norm-based U.S. population score, where the mean score ± standard deviation (SD) of 50 ± 10 is the normative population score (5). A score below 50 indicated worse HRQOL than the normative general population, while every 10 points represented one SD (8). For each SF-36 scale, we calculated the Z score by subtracting the mean scale score of the normative population stratified by the 1998 U.S. normal population (Z: mean, 0; SD, 1). A Z score value of 0 corresponds to the mean value of the normal population, and a Z score value of −1 or −2 corresponds to one or two SD below the normal population, respectively (19). Compared with the U.S. normative scores, Z score was preferred to the 0–100 based scoring algorithm because it provides a basis for meaningful comparison across scales and for easier interpretation (17). We defined Z score as a significantly impaired SF-36 domain with a subscale Z score ≤ 0.

### Statistical analysis

All calculations were performed using the SPSS 14.0 statistical software package (Statistical Package for Social Science, Chicago, IL, USA). Continuous variables were expressed as means with corresponding standard deviations, and categorical variables were expressed as numbers and percentages. The proportions were compared using the χ2 test. If the data were normally distributed, two group comparisons were performed with unpaired or paired, two-tailed *t*-test for means. If the data were not normally distributed, the nonparametric two-sided Mann–Whitney U test was used. Correlations were assessed using either Pearson or Spearman rank, as appropriate. ANOVA tests were used for multiple comparisons to compare the normative mean score of the different disease types (PAH, LHD-PH, CLD-PH, and CTEPH). Effect sizes were calculated as Cohen's d, modified using unpaired *t*-tests for unequal group sizes with Hedges' g equation and d converted from partial η^2^ in F tests (20). For all analyses, *p* < 0.05 were considered statistically significant. No α correction was performed for multiple tests, which could have led to the assumption of five of 100 false-positive tests in an exploratory setting (20).

Cox proportional hazards regression was used for determining risk factors for mortality at short-term, midterm, and long-term follow-ups adjusted by gender and age. The prognostic values of the parameters were tested using univariate Cox proportional hazards regression analysis at each follow-up, such as clinical data (diagnosis of PAH, WHO FC III and IV, decrease in Δ6MWD, increased ΔNT-proBNP), baseline hemodynamic variables (increase in mPAP, RAP, and PVR, and decrease in CI), decrease in ΔSF-36 in each domain, ΔPCS, and ΔMCS. Variables were all incorporated into the forward stepwise multivariable Cox proportional hazards model if confounders were considered in the univariate analyses or the variables have clinical importance. A *p*-value of < 0.05 was considered statistically significant.

## Results

### Patient clinical characteristics

HRQOL was examined in 559 consecutive adult inpatients (≥18 years) diagnosed with PH by RHC using SF-36 questionnaires. The baseline demographics and physical characteristics of the study patients are provided in Table 1. Main types of PH included are as follows: 49% PAH (group 1), 23% chronic thromboembolic PH (CTEPH, group 4), 20% PH due to chronic lung disease (CLD-PH, group 3), 5% PH due to left heart disease (LHD-PH, group 2), and 3% others. The patient population comprised predominantly female patients (67%), had WHO FC III and IV (74%), and had a mean age of 52 years with impaired exercise capacity and severe hemodynamic status. The sociodemographic characteristics of study patients are presented in Table 2. A majority of the patients were married, and only 10% lived alone. Approximately one-third of the patients have retired, but most patients can take care of themselves and contribute to part of their medical expenditure.

**Table 1:** Baseline demographic and clinical characteristics of patients with main pulmonary hypertension classification.

**Variable**	**PAH (*n* = 275)**	**LHD-PH (*n* = 28)**	**CLD-PH (*n* = 110)**	**CTEPH (*n* = 128)**	**All (*n* = 559)**
Age at diagnosis, years	45 ± 16	58 ± 18	62 ± 11	59 ± 15	52 ± 17
Female, *n* (%)	213 (78)	14 (50)	52 (47)	83 (65)	373 (67)
**WHO FC**, ***n*** **(%)**
I and II	71 (26)	10 (36)	17 (15)	39 (31)	143 (26)
III	172 (63)	16 (57)	74 (67)	87 (68)	358 (64)
IV	32 (12)	2 (7)	19 (17)	2 (2)	58 (10)
6MWD, meters*	338 ± 275	281 ± 155	236 ± 135	310 ± 139	305 ± 220
NT-proBNP, ng/mL	1,655 ± 2,652	1946 ± 2,733	1,133 ± 1,331	1,291 ± 1,711	1,513 ± 2,368
**Hemodynamics** ^**#**^				
RAP, mmHg	4 (1, 7)	7 (4, 9)	3 (1, 5)	2 (1, 3)	3 (1, 6)
mPAP, mmHg	53 (43, 62)	29 (25, 36)	34 (31, 47)	44 (33, 52)	46 (34, 56)
PAWP, mmHg	6 (4, 8)	15 (10, 18)	7 (4, 9)	6 (5,9)	6 (4, 9)
CI, L/min/m^2^	2.5 (2.1, 3.3)	2.9 (2.6, 3.4)	3.3 (2.7, 3.9)	2.9 (2.4, 3.4)	2.7 (2.2, 3.5)
PVR, Wood units	12 (8,16)	2 (2, 4)	5 (4, 8)	7 (4, 8)	9 (5, 13)
S_v_O_2_, %	64 (58, 71)	63 (59, 73)	65 (59, 74)	69 (67, 71)	64 (59, 69)
**PAH specific therapy**, ***n*** **(%)**				
**Monotherapy**	135 (49)	6 (21)	50 (46)	68 (53)	265 (47)
ERA	45 (16)	0	5 (5)	19 (15)	72 (13)
PDE5i	90 (33)	5 (18)	45 (41)	47 (37)	190 (34)
Prostanoid	1 (0.4)	1 (4)	0	2 (2)	4 (2)
Combination therapy	113 (41)	1 (4)	4 (4)	44 (34)	164 (30)
No targeted treatment	23 (8)	21 (75)	56 (51)	16 (13)	125 (22)

Values are expressed as mean ± SD or medians (interquartile range) or n (%), unless otherwise stated.

^*^Data on 6MWD were available from baseline 371 individuals.

^#^Data on hemodynamics were available from baseline 486 individuals.

CI, cardiac index; CLD-PH, pulmonary hypertension due to chronic lung disease and/or hypoxia; CTEPH, chronic thromboembolic pulmonary hypertension; ERA, endothelin receptor antagonist; LHD-PH, pulmonary hypertension due to left heart disease; PAH, pulmonary arterial hypertension; PH, pulmonary hypertension; mPAP, mean pulmonary arterial pressure; 6MWD, 6-min walking distance; NT-proBNP, N-terminal fragment of pro-brain natriuretic peptide; PAWP, pulmonary artery wedge pressure; PDE5i, phosphodiesterase type 5 inhibitor; PVR, pulmonary vascular resistance; RAP, right atrial pressure; SvO_2_, mixed venous oxygen saturation; WHO FC, World Health Organization functional class.

**Table 2:** Baseline social characteristics of patients with main pulmonary hypertension classification.

**Variable**	**PAH (*n* = 275)**	**LHD-PH (*n* = 28)**	**CLD-PH (*n* = 110)**	**CTEPH (*n* = 128)**	**All (*n* = 559)**
**Marital status**, ***n*** **(%)**					
Single	41 (15)	3 (11)	1 (1)	7 (6)	54 (10)
Married or living together	222 (81)	23 (82)	101 (92)	116 (91)	477 (85)
Divorced/Widowed	12 (4)	2 (7)	8 (7)	5 (4)	28 (5)
**Level of education**, ***n*** **(%)**					
Primary school	34 (12)	6 (21)	41 (37)	41 (32)	126 (23)
Basic high school	91 (33)	12 (43)	40 (36)	47 (37)	196 (35)
Junior college	107 (39)	5 (18)	24 (22)	28 (22)	170 (30)
University/College	43 (16)	5 (18)	5 (5)	12 (9)	67 (12)
**Occupation**, ***n*** **(%)**					
Not available	90 (33)	7 (25)	17 (16)	23 (18)	143 (26)
Farmer	44 (16)	3 (11)	35 (32)	24 (19)	107 (19)
Worker	44 (16)	2 (7)	9 (8)	17 (13)	74 (13)
Cadres	36 (13)	4 (14)	6 (6)	7 (5)	54 (10)
Retire	61 (22)	12 (43)	43 (39)	57 (45)	181 (32)
**Self-care status**, ***n*** **(%)**					
Yes	129 (47)	15 (54)	32 (29)	67 (52)	250 (45)
Part	131 (48)	13 (46)	63 (57)	60 (47)	278 (50)
No	15 (6)	0 (0)	15 (14)	1 (1)	31 (6)
**Medical payment**, ***n*** **(%)**					
All charged	78 (28)	8 (29)	25 (23)	23 (18)	140 (25)
Part charged	189 (69)	19 (68)	78 (71)	102 (80)	400 (72)
Insurance	8 (3)	1 (4)	4 (4)	1 (1)	14 (3)
Non-charged	0	0	3 (3)	2 (2)	5 (1)
**Annual income per capita**, **RMB 10,000 Yuan**	4.1 ± 2.2	4.2 ± 1.7	3.7 ± 1.5	3.8 ± 1.9	4.0 ± 2.1

Values are expressed as mean ± SD or n (%), unless otherwise stated.

CLD-PH, pulmonary hypertension due to chronic lung disease and/or hypoxia; CTEPH, chronic thromboembolic pulmonary hypertension; LHD-PH, pulmonary hypertension due to left heart disease; PAH, pulmonary arterial hypertension; PH, pulmonary hypertension.

### SF-36 HRQOL scores in baseline

**Figure 2:**
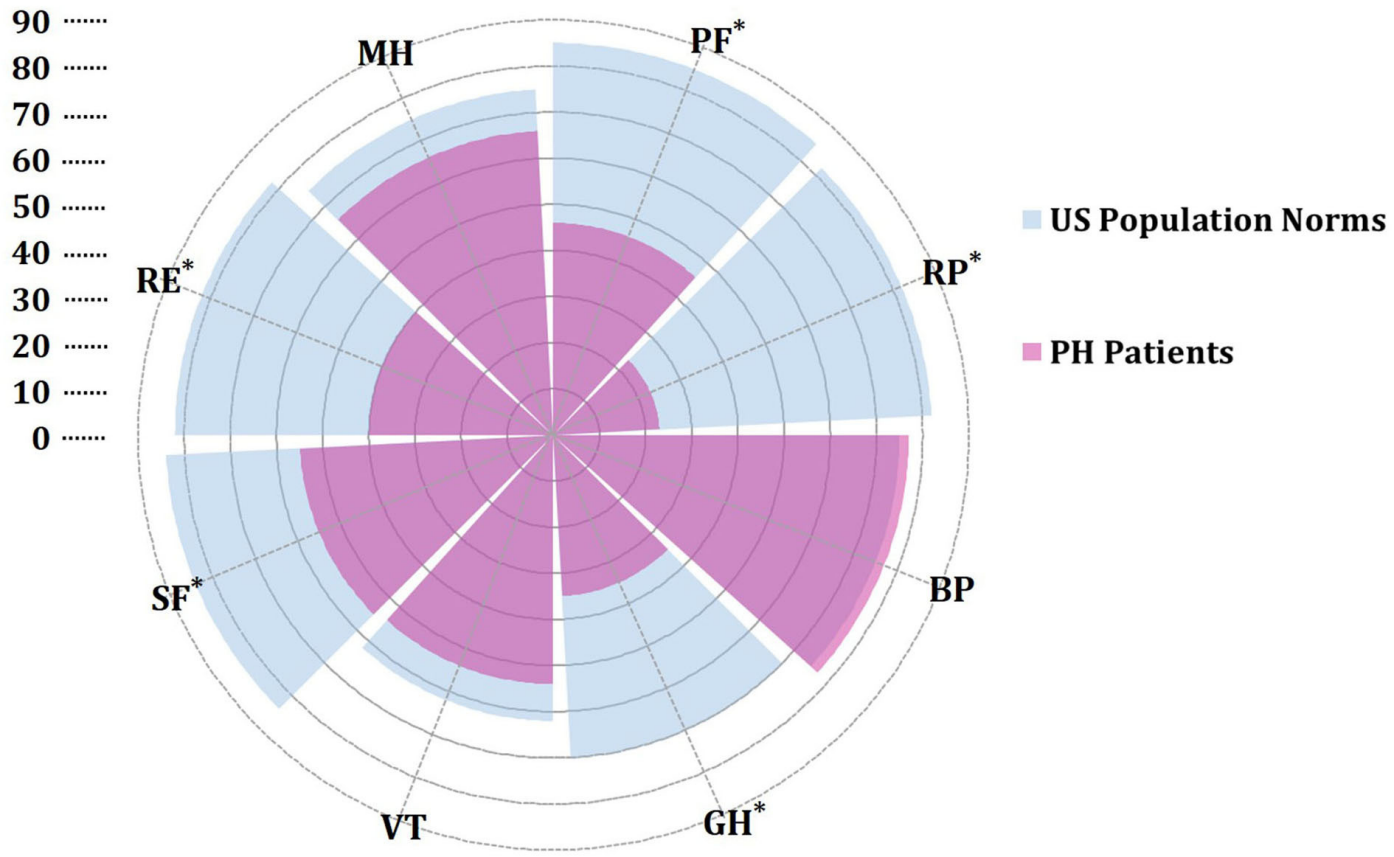
Rose radar plot of the individual domain in pulmonary hypertension (PH) vs. U.S. population normative values. All values are presented as means. *A *p* < 0.05 for comparison with U.S. population norms. Purple represents SF-36 scores in patients with PH. Blue represents SF-36 scores of U.S. population norms from the literature (21). BP, body pain; GH, general health; MH, mental health; PF, physical functioning; RE, role emotional; RP, role physical; SF, social functioning; VT, vitality.

**Figure 3:**
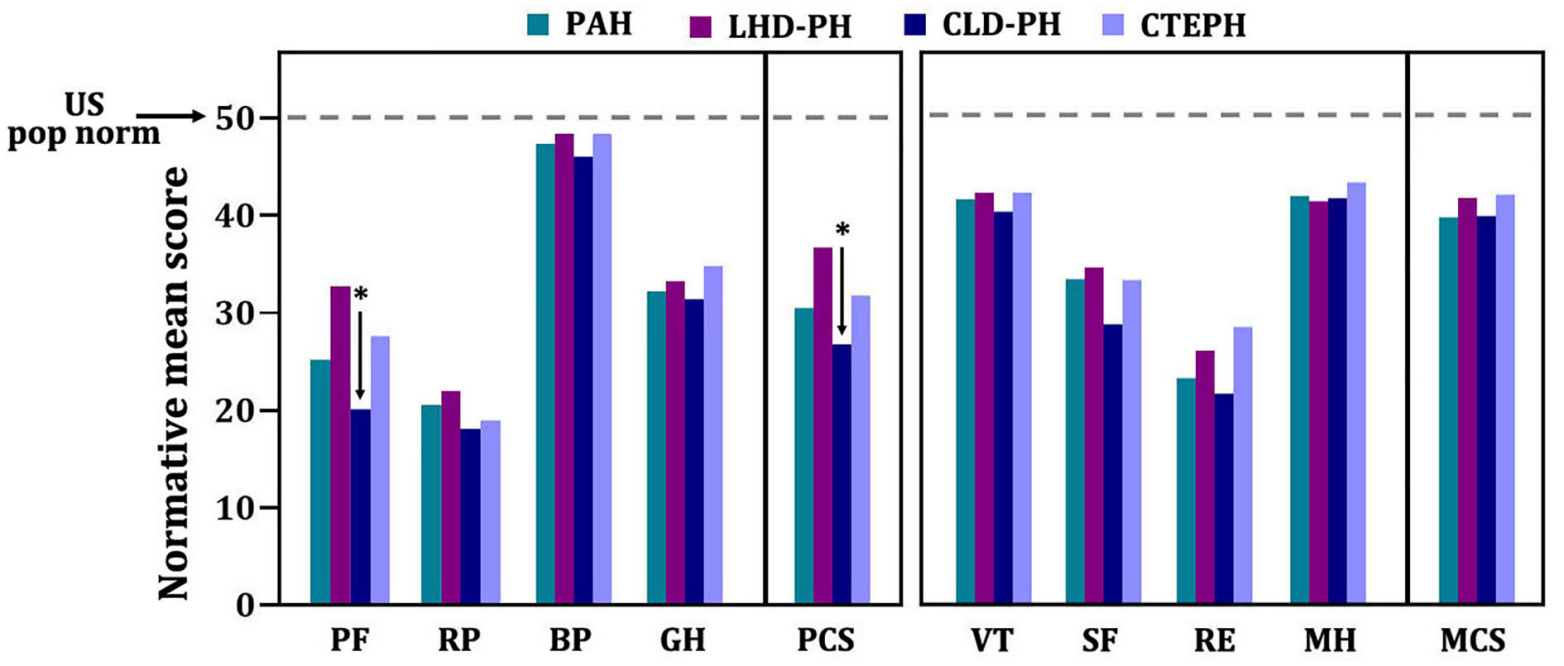
SF-36 scores of patients with pulmonary hypertension in different diagnosis types. Normative mean scores based on U.S. norm population are shown. Numerically higher scores represent better health-related quality of life. *A *p* < 0.05 indicates comparison among different diagnosis types in each domain or summary score. BP, body pain; GH, general health; MH, mental health; PF, physical functioning; RE, role emotion; RP, role physical; SF, social functioning; VT, vitality.

At baseline, the mean time between diagnosis by RHC and administration of the SF-36 was 13 days. HRQOL in patients with PH significantly differed from U.S. normative average values in five of eight domains (Figure 2). In the physical component summary, patients with PH had significantly less average scores in PF, RP, and GH subscales than the U.S. normative mean score, whereas in the mental component summary, SF and RE domains were significantly decreased. In addition, baseline PCS (30.3 ± 11.3) and the MCS (39.5 ± 15.1) have broadly impairments. We used a normative mean score (50 ± 10 SD)-based U.S. population to show the difference among the different disease types. All domain and summary scores in different diagnosis types were significantly lower than the U.S. normal population score of 50 in baseline, as shown in Figure 3. Patients with CLD-PH had significantly less PCS scores (26.8 ± 11.0) than those with different diagnosis types of PH, which could be due to its poor PF (20.4 ± 14.0) domain, whereas there was no difference of the MCS score in different diagnosis types of PH, despite impairment in each domain.

### Relation between baseline HRQOL and demographics and hemodynamics

**Figure 4:**
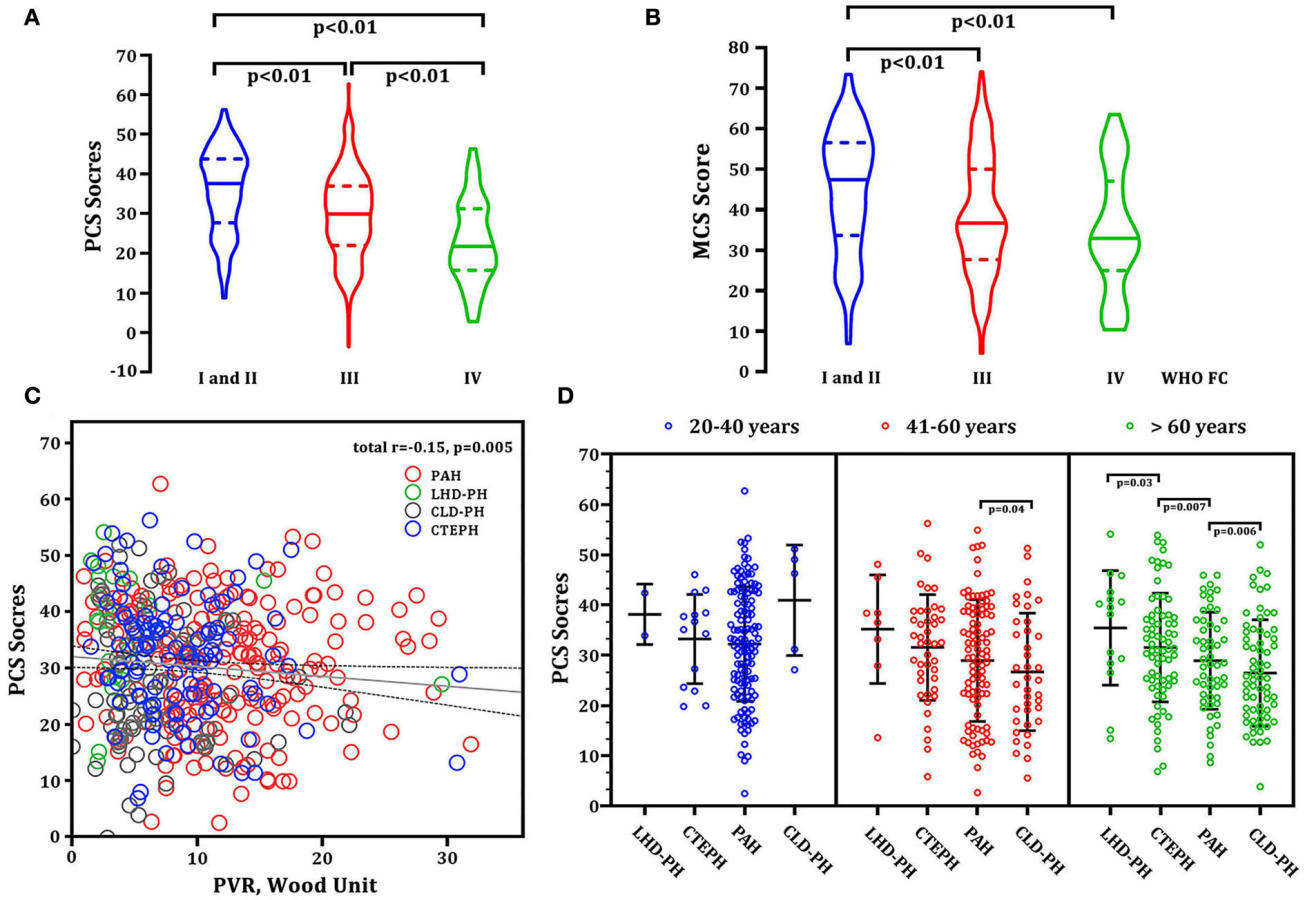
Correlations between baseline HRQOL and demographics and PVR. **(A)** PCS score vs. WHO FC severity; **(B)** MCS score vs. WHO FC severity; **(C)** PCS score vs. PVR (linear model: r^2^ = 0.15, *p* = 0.005). Red circles represent patients with PAH. Green circles represent the patients with LHD-PH. Gray circles represent CLD-PH. Blue circles represent CTEPH. **(D)** PCS scores at different ages and in different disease types. CLD-PH, pulmonary hypertension due to lung disease and/or hypoxia; CTEPH, chronic thromboembolic pulmonary hypertension; LHD-PH, pulmonary hypertension due to left heart disease; MCS, mental component summary; PAH, pulmonary arterial hypertension; PCS, physical component summary; PVR, pulmonary vascular resistance; WHO FC, World Health Organization functional class.

Baseline demographic characteristics such as age, gender, 6MWD, NT-proBNP, and hemodynamics (mPAP, CI, mRAP, and PAWP) were not significantly associated with each subscale, PCS or MCS. The baseline PCS score markedly decreased with the severity of WHO FC (Figure 4A). In part of MCS, patients with FC I and II had the highest MCS score. However, there was no difference between patients with WHO FC III and IV (Figure 4B). In addition, we only found the baseline PCS score was negatively associated with PVR in hemodynamics (r = −0.15, *p* = 0.005, Figure 4C). There was not any relation between the MCS score with hemodynamic parameters. Interestingly, the PCS score gradually decreased along with the growth of age. Patients older than 60 years das the worst PCS score than those aged 41–60 years and 20–40 years. Among the patients older than 60 years, patients with CLD-PH had significantly lower PCS scores than those with other diagnosis types (Figure 4D).

### Change trend in HRQOL

**Figure 5:**
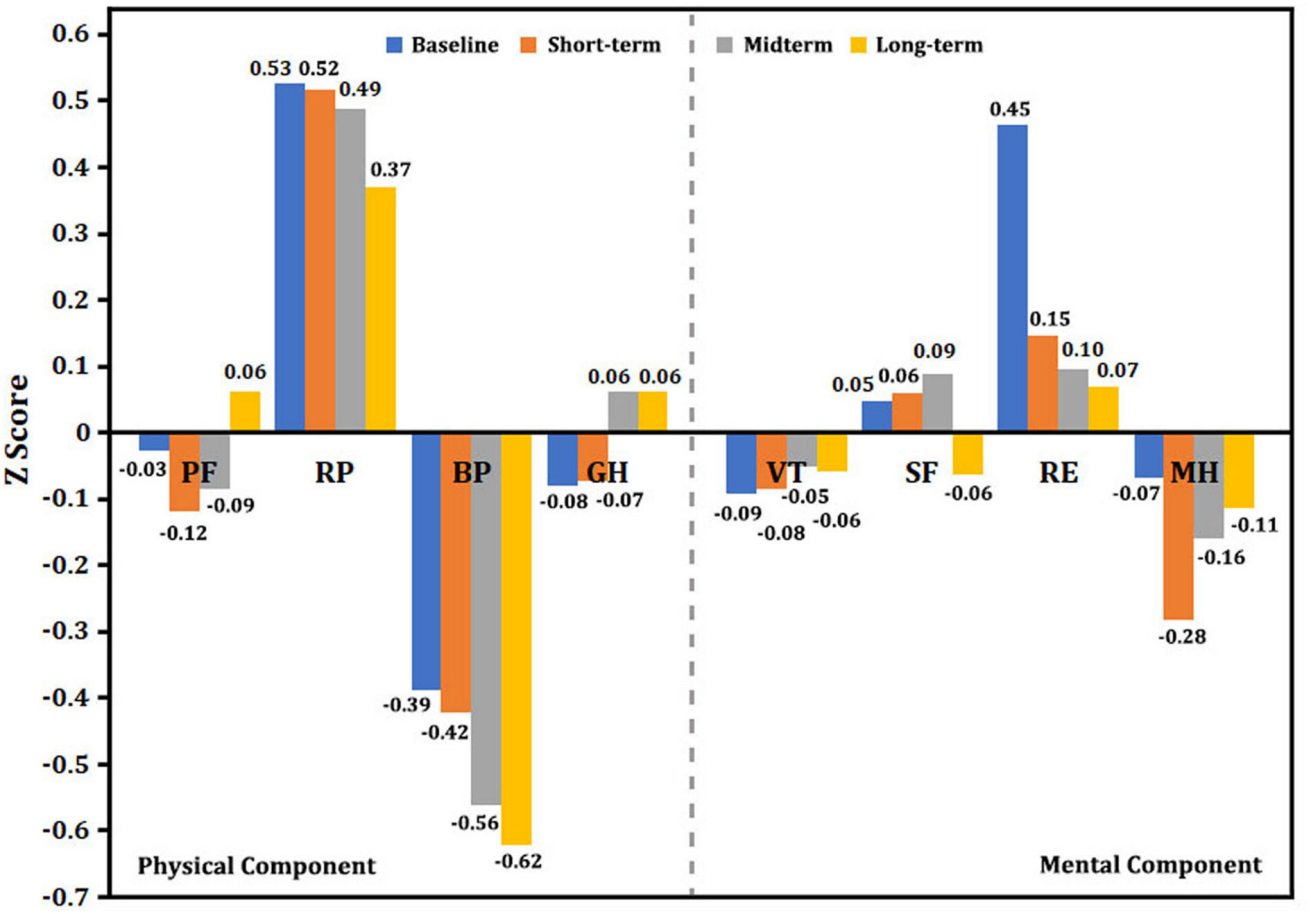
Single-scale norm-based Z score for the changing trend in SF-36. Normed by the 1998 U.S. general population (mean, 0; standard deviation, 1). A Z score < 0 indicates lower health status. The greater the absolute value of a negative number, the worse the health status. A higher positive value represents better health status. BP, body pain; GH, general health; MH, mental health; PF, physical functioning; RE, role emotion; RP, role physical; SF, social functioning; VT, vitality.

We used the Z score to interpret the changing trend in health status. As shown in Figure 5, the baseline physical component (PF, BP, and GH) and mental component (VT and MH) summary scores were significantly lower than the normative scores, but RP, SF, and RE were not lower than the mean score of norms. The long-term results of the Z score in the physical component summary showed that RP and BP tended to decline continuously, but PF and GH subscales significantly improved at the 5-year follow-up. Importantly, the Z score in the mental component seemed to persistently decrease from baseline to midterm and long-term follow-ups. Especially for SF and RE, the Z score was significantly reduced from 0.05 (baseline) to −0.06 (5-year follow-up) for SF as well as 0.45–0.07 for RE. At the end of the follow-up, there were 54 surviving patients, including 35 patients with PAH, nine patients with CLD-PH, and 10 patients with CTEPH who completed all questionnaires at each follow-ups. Similar results were observed in these patients, such as Z score of PF and GH significantly increased (see Supplementary Figures 1–4), whereas the Z scores of SF and RE showed a marked decrease from the baseline to long-term follow-up.

### Prognostic factor analyses

**Figure 6:**
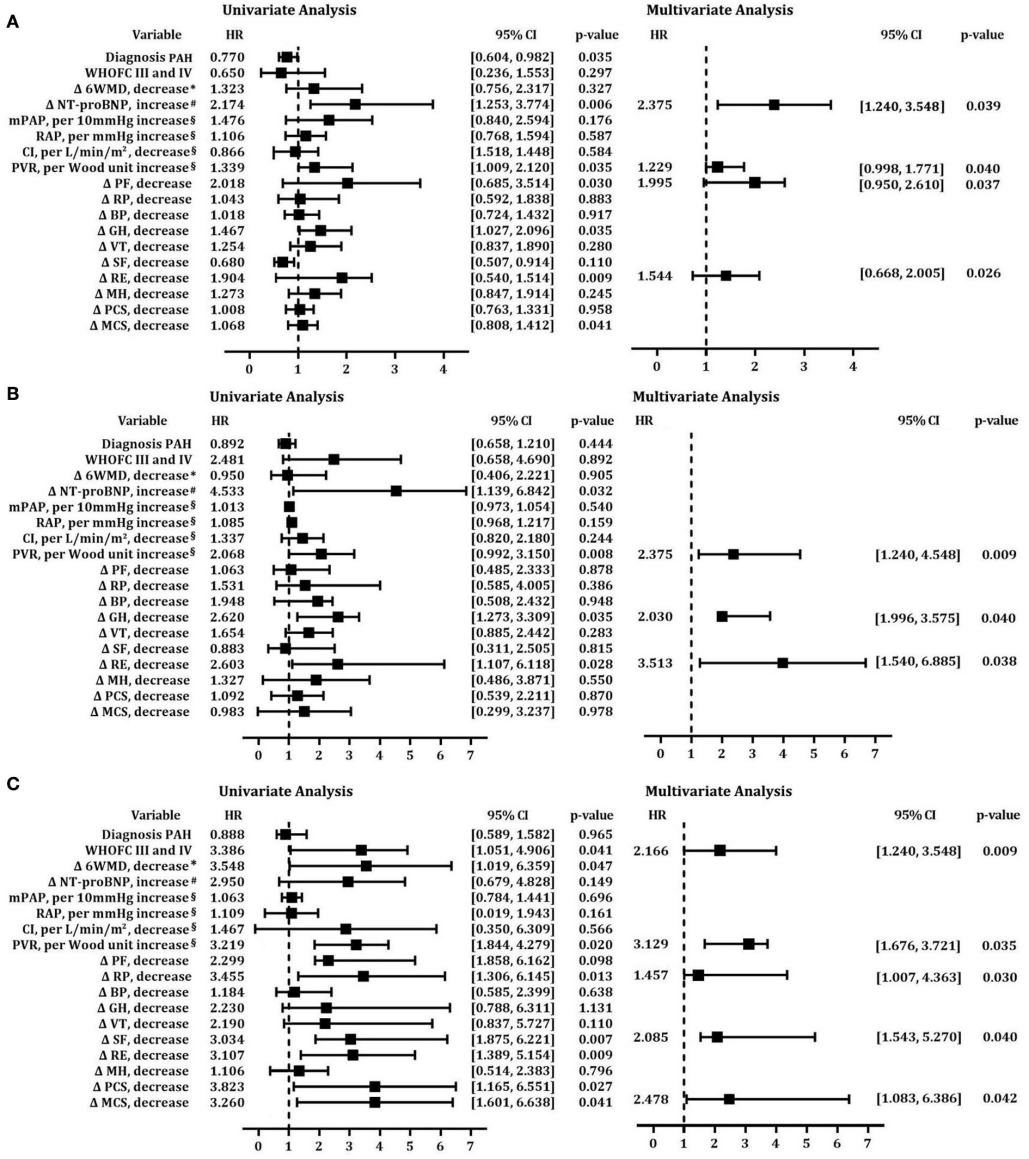
Cox proportional hazards predictors of mortality at short-term, midterm, and long-term follow-ups. Univariate and multivariate models of risk factors after adjustment for age at **(A)** short-term follow-up;**(B)** midterm follow-up; **(C)** long-term follow-up. BP, body pain; CI, cardiac index; GH, general health; MCS, mental component summary; MH, mental health; mPAP, mean pulmonary arterial pressure; 6MWD, 6-min walk distance; NT-proBNP, N-terminal fragment of pro-brain natriuretic peptide; PAH, pulmonary arterial hypertension; PCS, physical component summary; PF, physical functioning; PVR, pulmonary vascular resistance; RAP, mean right atrial pressure; RE, role emotion; RP, role physical; SF, social functioning; VT, vitality; WHO FC, World Health Organization functional class.

At each follow-up, we used univariate and multivariate Cox proportional hazards regression analyses to test risk factors for mortality adjusted by gender and age. In the short-term follow-up, increased NT-proBNP and baseline PVR, and decreased PF and RE are independent risk factors associated with mortality (Figure 6A). Accordingly, increased PVR, and decreased GH and RE are independent predictors of survival in multivariable analysis in the midterm follow-up (Figure 6B). Therefore, increased PVR and decreased RE at baseline are common risk factors in short-and midterm follow-ups. However, in the long-term follow-up, WHO FV III and IV, increased baseline PVR, and decreased RP, SF, and MCS had increased risk of mortality (Figure 6C).

## Discussion

PH is a progressive disease with high morbidity and mortality rates, despite advances in medical therapy. Patients with PH experienced debilitating symptoms, which had negative impacts on their HRQOL in terms of physical capability, psychological wellbeing, and social relations (20, 22). In this study, we found patients with PH tend to have severe impairments in both physical and emotional domains compared with those of U.S. norms. Patients with CLD-PH had significantly less baseline PCS scores, but there was no difference of MCS scores among the different diagnosis types of PH. Baseline WHO FC severity and PVR were correlated with the PCS score. At age >60 years, patients with CLD-PH had the worst PCS score, but those with CHD-PH had the highest PCS score in baseline. The PCS score was stable over 3 years of follow-up, but the MCS score tended to deteriorate from baseline to midterm and long-term follow-ups. Finally, Cox regression analysis demonstrated that increased baseline PVR, WHO FV III and IV, and decreased RP (physical component), social function (mental component), and MCS score had increased risk of mortality in the long-term follow-up. These results will provide additional knowledge about HRQOL in chronic disease management.

Despite the more prolonged survival of patients with PH, their HRQOL has been found to deteriorate as the condition progresses (11). Interestingly, in our study, only patients with CLD-PH had significantly less PCS scores (normative mean score 26.8 ± 11.0, *p* < 0.05) among the different diagnosis types of PH at baseline, which could be due to its poor PF (20.4 ± 14.0, *p* < 0.05) domain. There was no difference of PCS scores among PAH, CTEPH, and LHD-PH. The reasons for these differences are unclear but may be related to worst PF in the patients with CLD-PH. Compared with several other disease types, patients with CLD-PH might be older, have worse hemodynamics, and have relatively more underlying diseases or complications (23, 24). The PF subscale was used to examine a person's perceived limitation in performing any physical activity (18). Also, the decrease in the PF score is an independent risk factor associated with mortality in the short-term follow-up. The PF domain reflected a wide range of physical disabilities that caused significant changes in family life, social interactions, and financial situations (10, 18). In fact, the economic burden of patients is substantial at the beginning of diagnosis PH in China (25). The PF did not always decline as treatment progressed and QOL improved, rather it was better at the long-term follow-up. On the contrary, the value of RP and BP continued to decrease over the entire study period. A total of three subscales (PF, RP, and BP) were found to correlate most highly with the physical component and contribute most to the scoring of the PCS measure (26). However, the PCS score was not included in the Cox predictive model in this study. Halimi et al. reported that the PCS score was poor but stable over the 3 years of follow-up in patients with PAH (10). Therefore, the changing trend of physical component summary score was heterogeneous, highlighting the complex relation between patient perception and disease pathogenesis.

The self-assessment of HRQOL is a cognitive process, and psychological factors can easily influence it (27, 28). PAH and CTEPH impacts involving poor mental and psychological factors have been repeatedly demonstrated (14, 15, 29). In our study, we did not find any differences between the MCS score and each domain in different diagnosis types of the disease, despite the worst PCS score in patients with CLD-PH. Meanwhile, there was no significant correlation between the MCS score and hemodynamics. These results suggested that patients did not realize severity of the disease at the initial stage of diagnosis. However, the change of the mental component summary score persistently declined from baseline to the long-term follow-up. Importantly, decreased RE was an independent predictor of mortality in the short-term and midterm follow-ups. In the long-term follow-up, the predictor of survival changed to SF and MCS. The occurrence of mental disorders has been linked to problems in daily activities. Due to the type and severity of PH, patients were vulnerable to emotional problems, including anxiety and depression (14, 30). Moderate to severe depression accounted for 20%−50% in patients with PAH (31, 32). Accordingly, depression and anxiety correlated with most SF-36 scales in CTEPH, and depression was more frequent in patients with CTEPH (56%) than in patients with PAH (30%) (12, 13). A study of Chinese survey indicated mental disorders were associated with the financial situation and right ventricular enlargement, and PAH and CTEPH patients with depression had a worse prognosis (14). Unexpectedly, the SF scale was incorporated into Cox multiple modeling to replace the RE scale. RE and SF have been shown to be most responsive in comparison to the change in the severity of depression, as well as drug treatment and interpersonal therapy for depression (26, 33). Compared with the RE scale, SF represented more extreme and frequent interference with normal social activities due to physical and emotional problems (26). The present study revealed self-assessment of the mental condition might gradually deteriorate. The patients' point of view regarding their health status is essential in decision-making procedures. There should be more randomized controlled trials to address the role of interventions including psychological consultation (34).

## Study limitations

This study has several limitations. First, this is a single -center retrospective study. Although the sample size of this study is larger than that of most studies, the size of LHD-PH is not large enough to provide sufficient patient numbers. Second, SF-36 is not a disease-specific HRQOL tool like the Cambridge Pulmonary Hypertension Outcome Review (CAMPHOR), which exhibited superior psychometric properties compared with the SF-36 in the assessment of PH patient-reported outcomes (35). In addition, the follow-up assessments were not standardized and lost to RHC testing, which made it difficult to analyze the relation between the change of hemodynamic parameters and HQOL. Finally, despite the median interval between diagnosis and follow-up being almost 1, 3, and 5 years, it was unavoidable to be biased toward the time–effect test.

## Conclusion

The patients' point of view regarding their health status is essential in decision-making procedures. In conclusion, the present study documented that patients with PH tend to have impaired HQOL in both physical and mental components using the generic SF-36. Measuring how patients “feel, function, or survive” is a clinically meaningful endpoint. Notably, some subscale scores in the physical component improved; however, the changing trend of the mental condition continuously declined from the baseline to long-term follow-up. Therefore, identifying and intervening mental progresses is a major issue in PH management.

## Data availability statement

The raw data supporting the conclusions of this article will be made available by the authors, without undue reservation.

## Ethics statement

The studies involving human participants were reviewed and approved by Ethic Committee of Shanghai Pulmonary Hospital (Number: K16-293). The patients/participants provided their written informed consent to participate in this study.

## Author contributions

LW and RZ contributed to the study design, study conduct and supervision, scientific overview, data analysis, and editing of the manuscript. J-LL and FX contributed to patient enrollment, data analysis, scientific interpretation, and drafting and editing the original manuscript. H-TLiu, H-TLi, Q-HZ, C-YS, YZ, LY, W-YW, HL, S-GG, RJ, and J-ML contributed to recruitment of participants, data collection and curation, and formal analysis. FX contributed as a statistician. All authors participated in the design of the study and/or patient enrollment, and meet criteria for authorship, reviewed the manuscript, and approved the final version for submission.

## Funding

The study was supported in part by the National Natural Science Foundation of China (82000059) (to LW), the National Natural Science Foundation of Shanghai Scientific and Technological Committee (22ZR1452600) (to RZ), and the Shanghai Pujiang Program (2021PJD060) (to LW).

## Conflict of interest

The authors declare that the research was conducted in the absence of any commercial or financial relationships that could be construed as a potential conflict of interest.

## Publisher's note

All claims expressed in this article are solely those of the authors and do not necessarily represent those of their affiliated organizations, or those of the publisher, the editors and the reviewers. Any product that may be evaluated in this article, or claim that may be made by its manufacturer, is not guaranteed or endorsed by the publisher.

## Abbreviations

BP: body pain
CI: cardiac index
95% CI: 95% confidence interval
CLD-PH: pulmonary hypertension due to lung disease and/or hypoxia
CTEPH: chronic thromboembolic pulmonary hypertension
GH: general health
LHD-PH: pulmonary hypertension due to left heart disease
MCS: mental component summary
MH: mental health
mPAP: mean pulmonary arterial pressure
6MWD: 6-min walk distance
NT-proBNP: N-terminal fragment of pro-brain natriuretic peptide
PAH: pulmonary arterial hypertension
PAWP: pulmonary artery wedge pressure
PCS: physical component summary
PF: physical functioning
PVR: pulmonary vascular resistance
RAP: mean right atrial pressure
RE: role emotion
RP: role physical
SF: social functioning
SF-36: Medical Outcomes Study 36-Item Short Form Health Survey
VT: vitality
WHO FC: World Health Organization functional class.
