# Supplementary material for: Long-term outcomes in health-related quality of life influence chronic disease management in patients with pulmonary hypertension
Source: Front Cardiovasc Med. 2022 Nov 10;9:1008253. doi: 10.3389/fcvm.2022.1008253 (PMC9685316; doi:10.3389/fcvm.2022.1008253)
Supplement: Supplementary file 1 [file Data_Sheet_1.DOCX]

**
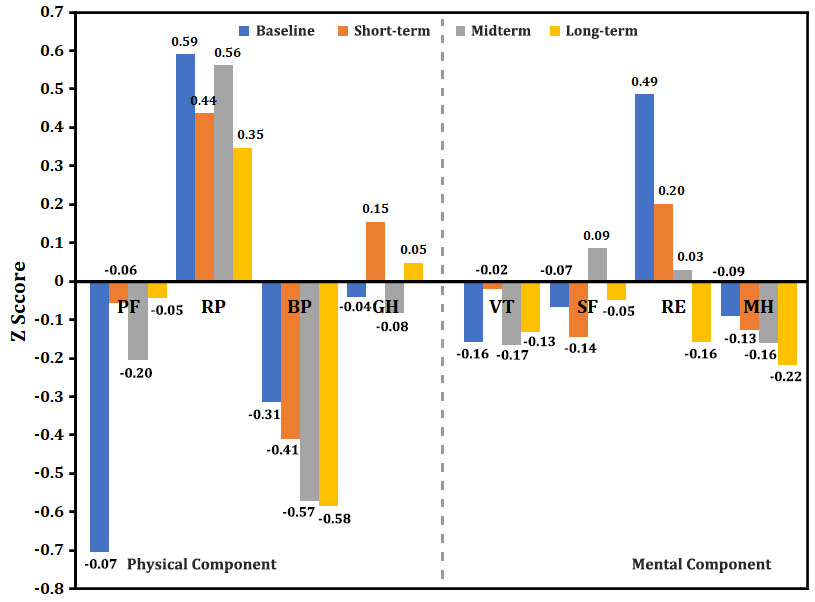
**

**Supplementary** **Figure 1. Single-scale norm-based Z Score for change trend in patients with PAH.**

Normed by 1998 US general population (mean, 0; standard deviation, 1). Z score< 0 indicated lower health status. The greater the absolute value of a negative number, the worse for health status. A higher positive value represented better for health status.

BP: body pain; GH: general health; MH: mental health; PAH: pulmonary arterial hypertension; PF: physical functioning; RE: role emotion; RP: role physical; SF: social functioning; VT: vitality;


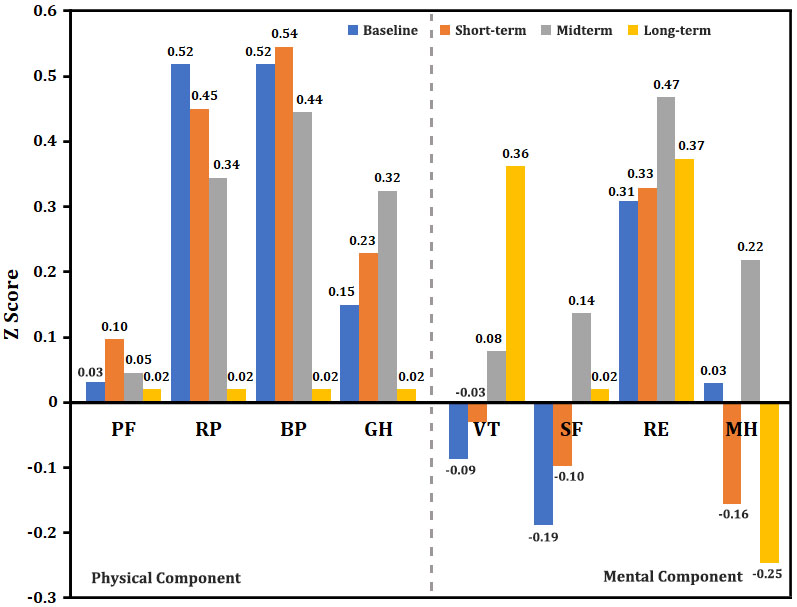


**Supplementary Figure 2. Single-scale norm-based Z Score for change trend in patients with CLD-PH.**

Normed by 1998 US general population (mean, 0; standard deviation, 1). Z score< 0 indicated lower health status. The greater the absolute value of a negative number, the worse for health status. A higher positive value represented better for health status.

BP: body pain; CLD-PH: pulmonary hypertension due to lung disease and/or hypoxia; GH: general health; MH: mental health; PAH: pulmonary arterial hypertension; PF: physical functioning; RE: role emotion; RP: role physical; SF: social functioning; VT: vitality;


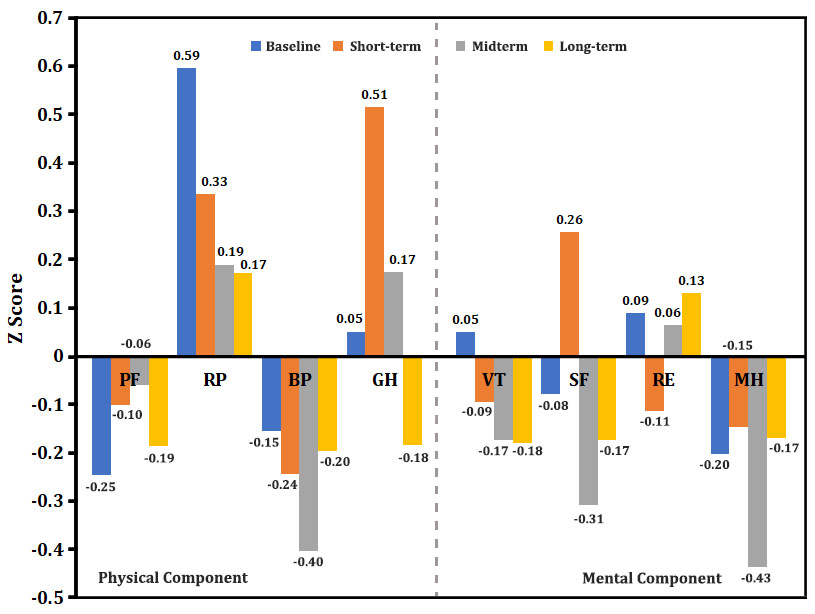


**Supplementary Figure 3. Single-scale norm-based Z Score for change trend in patients with CTEPH.**

Normed by 1998 US general population (mean, 0; standard deviation, 1). Z score< 0 indicated lower health status. The greater the absolute value of a negative number, the worse for health status. A higher positive value represented better for health status.

BP: body pain; CTEPH: chronic thromboembolic pulmonary hypertension; GH: general health; MH: mental health; PAH: pulmonary arterial hypertension; PF: physical functioning; RE: role emotion; RP: role physical; SF: social functioning; VT: vitality;

**
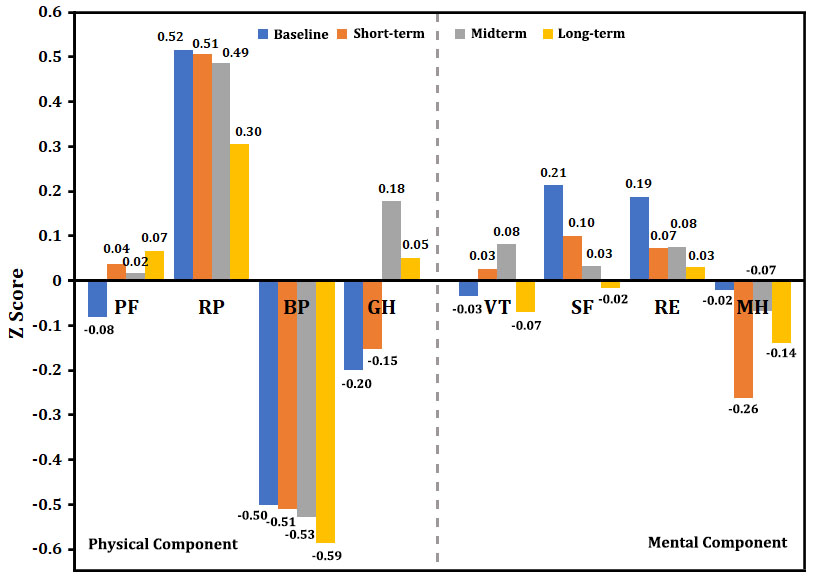
**

**Supplementary Figure 4. Single-scale norm-based Z Score for change trend in survivors at last follow-up.**

Normed by 1998 US general population (mean, 0; standard deviation, 1). Z score< 0 indicated lower health status. The greater the absolute value of a negative number, the worse for health status. A higher positive value represented better for health status.

BP: body pain; GH: general health; MH: mental health; PAH: pulmonary arterial hypertension; PF: physical functioning; RE: role emotion; RP: role physical; SF: social functioning; VT: vitality;
